# Additional Routes to *Staphylococcus aureus* Daptomycin Resistance as Revealed by Comparative Genome Sequencing, Transcriptional Profiling, and Phenotypic Studies

**DOI:** 10.1371/journal.pone.0058469

**Published:** 2013-03-15

**Authors:** Yang Song, Aileen Rubio, Radheshyam K. Jayaswal, Jared A. Silverman, Brian J. Wilkinson

**Affiliations:** 1 Microbiology Group, School of Biological Sciences, Illinois State University, Normal, Illinois, United States of America; 2 Cubist Pharmaceuticals, Inc., Lexington, Massachusetts, United States of America; University of Liverpool, United Kingdom

## Abstract

Daptomycin is an extensively used anti-staphylococcal agent due to the rise in methicillin-resistant *Staphylococcus aureus*, but the mechanism(s) of resistance is poorly understood. Comparative genome sequencing, transcriptomics, ultrastructure, and cell envelope studies were carried out on two relatively higher level (4 and 8 µg/ml^−1^) laboratory-derived daptomycin-resistant strains (strains CB1541 and CB1540 respectively) compared to their parent strain (CB1118; MW2). Several mutations were found in the strains. Both strains had the same mutations in the two-component system genes *walK* and *agrA*. In strain CB1540 mutations were also detected in the ribose phosphate pyrophosphokinase (*prs*) and polyribonucleotide nucleotidyltransferase genes (*pnpA*), a hypothetical protein gene, and in an intergenic region. In strain CB1541 there were mutations in *clpP*, an ATP-dependent protease, and two different hypothetical protein genes. The strain CB1540 transcriptome was characterized by upregulation of *cap* (capsule) operon genes, genes involved in the accumulation of the compatible solute glycine betaine, *ure* genes of the urease operon, and *mscL* encoding a mechanosensitive chanel. Downregulated genes included *smpB*, *femAB* and *femH* involved in the formation of the pentaglycine interpeptide bridge, genes involved in protein synthesis and fermentation, and *spa* encoding protein A. Genes altered in their expression common to both transcriptomes included some involved in glycine betaine accumulation, *mscL*, *ure* genes, *femH*, *spa* and *smpB*. However, the CB1541 transcriptome was further characterized by upregulation of various heat shock chaperone and protease genes, consistent with a mutation in *clpP*, and *lytM* and *sceD*. Both strains showed slow growth, and strongly decreased autolytic activity that appeared to be mainly due to decreased autolysin production. In contrast to previous common findings, we did not find any mutations in phospholipid biosynthesis genes, and it appears there are multiple pathways to and factors in daptomycin resistance.

## Introduction

Increases in the number of methicillin-resistant *Staphylococcus aureus* (MRSA) strains, increases in vancomycin minimum inhibitory concentrations (MICs) in such strains resulting in vancomycin-intermediate *S. aureus* (VISA) and vancomycin-resistant *S. aureus* (VRSA), have provided impetus to the development of novel antistaphylococcal antimicrobial agents [1]. Daptomycin is an acidic lipodepsipeptide natural product with high activity against gram-positive bacteria [2]. It has been approved for the treatment of complicated skin and soft tissue infection, *S. aureus* bacteremia and *S. aureus* right-sided endocarditis in the US [3]. Daptomycin is a significant component of the anti-staphylococcal armamentarium and is an extensively used therapeutic agent.

The antibacterial activity of daptomycin is dependent on calcium [4], and the agent is believed to interact with the membrane via its lipophilic tail. Daptomycin has been shown to cause membrane depolarization and leakage of intracellular metabolites and ions [5], [6]. Daptomycin has been proposed to oligomerize on the membrane and disrupt its functional integrity, or to insert into the membrane and cause positive membrane curvature [6]–[9]. Transcriptional profiling studies of the response to daptomycin have shown induction of the cell wall stress stimulon including its critical two-component regulator *vraSR* in *S. aureus* and *liaSR* in *Bacillus subtilis*, and genes responsive to membrane depolarization [10], [11]. Recent work by Pogliano et al. [12] has shown that insertion of daptomycin into the membrane results in the formation of membrane patches that redirect the localization of proteins involved in cell division and cell wall synthesis, thereby allowing reconciliation of previous observations on the mode of action of daptomycin involving both membrane depolarization and inhibition of cell wall synthesis.

Given the extensive use of daptomycin in treatment of infectious diseases it is important to understand mechanisms of resistance against the agent. It has proved possible to select strains showing decreased daptomycin susceptibility/daptomycin resistance in vitro by cultivation in increasing concentrations of the agent [13]. Also, daptomycin-resistant strains have been isolated from patients undergoing daptomycin therapy [14]–[17]. Furthermore, VISA may show decreased susceptibility to daptomycin [18]–[21].

The mechanism of daptomycin resistance has been studied and various observations have been made, but there is no one consensus mechanism of daptomycin resistance. Changes in membrane potential or the ability of daptomycin to depolarize the membrane have been observed in resistant strains [13], [22]–[24]. Various changes in the cytoplasmic membrane in daptomycin-resistant strains have been described. Jones et al. [22] reported on a clinical daptomycin resistant strain and found it had increased membrane fluidity, increased translocation of lysyl-phosphatidyl glycerol to the outer half of the cytoplasmic membrane bilayer, and the cells had an increased positive charge compared to a susceptible parent strain. Mishra et al. [25] reported on laboratory-derived daptomycin resistant strains and they found increased lysyl-phosphatidyl glycerol and increased flipping of this phospholipid to the outer half of the bilayer, increased expression of *mprF*, a gene encoding an enzyme responsible for adding lysine to phosphatidyl glycerol forming the positively charged species lysylphosphatidyl glycerol [26], [27]. Also the *dlt* operon, which is responsible for adding D-alanine residues to teichoic acid thereby reducing its negative charge, was increased in expression. However, in contrast to the findings of Jones et al. [22] membrane fluidity was decreased, and despite increased *mprF* and *dlt* operon expression, cell positive charge was not increased in the report of Mishra et al. [25]. Nevertheless, in a later paper [28] studying a new day 20 serial in vitro passage daptomycin resistant strain small changes in lysylphosphatidyl glycerol features, increased carotenoid concentrations, decreased membrane fluidity and different mutations were observed compared to previous day 20 isolates [25]. However, Mishra et al. [29] have reported increased membrane fluidity in isogenic pairs of clinical MRSA daptomycin-resistant strains that were also cross-resistant to host defense cationic antimicrobial peptides.

Comparative genome sequencing of laboratory-derived daptomycin-resistant strains [30] revealed mutations in *mprF*, *rpoB* (RNA polymerase subunit B), and *walK*, the sensor component of a two-component system playing a critical role in *S. aureus* cell wall metabolism [31]. Daptomycin susceptibility is increased in *mprF* knockout strains, and when *mprF* expression is decreased using an *mprF* antisense plasmid [32]. In those daptomycin-resistant strains where mutations in *mprF* have been detected it is believed they are gain of function mutations [32]. However *mprF* mutations are not found in all daptomycin-resistant strains [15], [33].

Cui et al. [34] carried out whole genome sequencing of a laboratory-derived daptomycin resistant strain 10*3d1 previously described by Camargo et al. [35]. Five mutations were found, one in a hypothetical protein gene, mutations in genes *rplV*, *rplC* encoding ribosomal proteins, and a mutation in *rpoB*. Replacement of intact *rpoB* in the parent strain with mutated *rpoB* conferred the phenotype of reduced daptomycin susceptibility, the MIC going from 0.25 to 1.5 µg ml^−1^. However, mutations in *rpoB* have not been found in all strains studied. In order to attain relatively high daptomycin MICs strains typically have mutations in more than one gene, and mutations are not found in the same genes in all strains [15], [18], [30], [33].

In this paper we report on genomic, transcriptomic, ultrastructure, and cell wall autolysis studies of two strains, CB1540 and CB1541, with relatively high daptomycin MICs (CB1541 4 µg ml^−1^and CB1540 8 µg ml^−1^), derived from methicillin-resistant parent strain MW2 (CB1118) by serial passage. Neither of the strains have mutations in *mprF* or *rpoB*, but both are mutated in *walK* and *agrA*, and other genes. The transcriptomes of the strains showed some common features and some differences. Both strains showed significantly decreased autolytic activity.

## Results and Discussion

### Initial Characterization of The Strains

The daptomycin MIC for strains CB1540 and CB1541 were 8 µg ml^−1^, and 4 µg ml^−1^ respectively compared to 1 µg ml^−1^ for parent strain CB1118 (Table 1). The MIC for vancomycin was increased from 0.5 µg ml^−1^ to 2 µg ml^−1^ in strains CB1540 and CB1541. However, the nafcillin MIC was decreased from 16 µg ml^−1^ in the parent to 4 µg ml^−1^ in the two daptomycin-resistant strains. Sensitization of daptomycin resistant strains to β-lactam antibiotics has been referred to as the “seesaw effect” [36] and has been observed in heterogeneous but not homogenous MRSA strains [37]. The underlying mechanism of the see saw effect is unclear. On agar plates, the colonies of CB1540 and CB1541 were white in color (Table 1), indicating a lower carotenoid content, and significantly smaller than those of CB1118. In liquid culture the strains grew much slower than the parent strain with mean generation times of 47 min and 62 min for strains 1540 and 1541 respectively compared to 29 min for CB1118 (Table 1). Slow growth is often associated with decreased antibiotic susceptibility [38] including in VISA [39].

**Table 1:** Strains studied and antibiotic MICs.

Strain	Antibiotic MIC (ug ml^−1^)			Mean generation time (min)	%OD_465_ warm methanol extract
	Daptomycin	Vancomycin	Nafcillin		
CB1118 (MW2)	1	0.5	16	29	100
CB1540	8	2	4	47	54.4
CB1541	4	2	4	62	52.5

### Mutations in strains CB1540 and CB1541 compared to CB1118

These mutations present in the strain are summarized in Table 2.

**Table 2:** Single nucleotide polymorphisms in strains CB1540 and CB1541 compared to parent strain CB1118.

Strain	Locus ID	Gene Name	Product	Start	End	Mutation position	Nucleotide change	Amino acid change	Mutation position in protein
CB1540	MW0019	*walK*	two-component sensor histidine kinase	1	1827	28	C→T	L→F	9
	MW0455	*prs*	ribose-phosphate pyrophosphokinase	1	966	701	C→T	A→V	234
	MW1109		conserved hypotehtical protein	1	1647	398	T→A	L→H	133
	MW1157	*pnpA*	polyribonucleotide nucleotidyltransferase	1	2097	1037	T→C	L→P	346
	MW1963	*agrA*	accessory gene regulator A	1	717	300	C→A	Y→*	100
CB1541	MW0019	*walK*	two-component sensor histidine kinase	1	1827	28	C→T	L→F	9
	MW0730	*clpP*	ATP-dependent Clp protease proteolyticsubunit homologue	1	588	281	G→A	G→D	94
	MW1913		hypothetical protein	1	204	27	T→A	V→V	9
	MW1927		hypothetical protein	1	162	18	T→G	K→N	6
	MW1963	*agrA*	accessory gene regulator A	1	717	300	C→A	Y→*	100

#### *walk*

A change from a C to T at position 28 resulting in an amino acid change from leucine to phenylalanine was found in *walK* in both strains. This is not the same mutation as that reported by Friedman et al. [30]. Amino acid substitutions were not detected in any functional domains. WalK is the histidine kinase sensor of an essential two-component system that controls peptidoglycan metabolism through regulation of the expression of most of the peptidoglycan hydrolase genes [31].

#### *agrA*

There was a change from a C to A at position 300 resulting in an amino acid change from tyrosine to a nonsense mutation in *agrA* in both strains resulting in a prematurely terminated protein.

The *agr* locus in *S. aureus* encodes a quorum sensing system that controls the expression of virulence and other genes via a two-component system [40], [41]. AgrA is a response regulator that upregulates its own promoter, P2, in the *agr* locus, and the RNAIII promoter. RNAIII is the effector of the *agr* system. Several hundred genes have been identified to be upregulated by RNAIII [42]. Tsuji et al. [43] have noted an association with an increase in the vancomycin-intermediate phenotype in *agr-*defective strains. Also, *agr* mutants show decreased autolysis [20], [44].

#### *pnpA*

There was a mutation in position 1037 changing a T to a C and a leucine to proline in strain CB1540. Polynucleotide phosphorylase is a phosphorolytic exoribonuclease. In gram-negative enteric bacteria it has been shown that PnpA controls the expression of small non-coding RNAs. PnpA is involved in quality control of RNA precursors and plays roles in expression of virulence functions and growth resumption after cold shock [45]–[48]. There were extensive changes in transcription in strain 1540 and PnpA may be involved in these.

#### *prs*

A C was changed to a T in position 346 resulting in a change from an alanine to valine in strain CB1540. This gene encodes phosphoribosylpyrophosphate synthetase and a mutation in this gene may cause requirements for purine and pyrimidine nucleotides, consistent with the critical role of ribosyl pyrophosphate in the biosynthesis of these molecules. There was significant down-regulation of the purine biosynthetic operon in strain CB1540, perhaps reflecting increased reliance on exogenous purines.

#### *clpP*

There was a mutation in the gene encoding ClpP protease in strain CB1541 at position 94 from a G to an A changing a glycine to aspartic acid. *clpP* is involved in protein quality control degrading abnormal proteins accumulating in stress conditions. Michel et al. [49] have shown that ClpP in *S. aureus* has a global regulatory impact on various regulons including the heat shock response, oxidative stress response, autolysis and DNA repair. Study of a Δ*clpP* mutant showed decreased expression of *agr* and *agr*-dependent virulence factors, and increased expression of heat shock regulon genes controlled by CtsR and HrcA. The transcriptional profile of strain CB1541 is characterized by significant upregulation of genes encoding chaperones and proteases. However, McGillvray et al. [50] have recently described chemical inhibition of *S. aureus* ClpXP by a pharmacological inhibitor, F2. F2 treatment sensitized *S. aureus* to cell envelope-active antibiotics including daptomycin. Including strains show decreased susceptibility to daptomycin.

In addition there were mutations in one gene for a hypothetical protein in CB1540 and two hypothetical protein genes in strain CB1541 (Table 2).

### Mutations Identified in Other Studies of Daptomycin-Resistant Strains

A variety of mutations have been detected in daptomycin-resistant strains. In the first study of this Friedman et al. [30] using complete-genome comparisons (CGS) on various laboratory-derived strains detected mutations resulting in amino acid substitutions in MprF, sensor kinase WalK, and the β and β′ chains of RNA polymerase RpoB and RpoC. In addition intergenic mutations were detected. However, Pillai et al. [33] detected no amino acid substitutions in MprF in clinical *S. aureus* isolates that developed vancomycin heteroresistance and daptomycin nonsusceptibility. Cui et al. [34] have shown that introduction of a mutation in *rpoB* into a vancomycin-susceptible *S. aureus* VSSA conferred resistance to both vancomycin and daptomycin on the resulting strain.

Recently, Peleg et al., [51] have reported the results of whole genome sequencing on 21 clinical and 12 laboratory strains that developed daptomycin-nonsusceptibility after exposure to daptomycin compared to their daptomycin susceptible parent strains. The MICs of the nonsusceptible strains were 2 or 4 ug daptomycin ml^−1^. The laboratory strains had an average of two mutations in coding regions compared to six in clinical strains. We found six mutations in our laboratory-derived strains, which had higher daptomycin MICs than those studied by Peleg et al. [51]. One hundred percent of the strains studied by Peleg et al. [51] had mutations in phospholipid biosynthesis genes, including mutations in the previously identified *mprF* and other phospholipid biosynthesis genes that were not found in our strains. Mehta et al. [52] recently reported various point mutations in *mprF* in a group of clinical MRSA strains. Varying percentages of the strains studied by Peleg et al. [51] had mutations in genes in other gene categories including cellular processes, transporters, protein synthesis, regulatory functions and two-component systems. As in our study, Peleg et al. [51] noted mutations in *walK* and *agrA* in some strains, and in *clpX* in some strains whereas we noted a mutation *clpP* in strain CB1541. It is possible that mutations in various genes may result in slow growth in daptomycin-resistant mutants.

### Mutations Identified in VISA Strains

As pointed out above VISA may show decreased daptomycin susceptibility [18]–[21]. Strains CB1540 and CB1541, which were selected for decreased daptomycin susceptibility, also show decreased vancomycin susceptibility. Hence, it is likely of interest to see what mutations have been reported in VISA strains. This information has been reviewed by Howden et al. [53]. Mutations in genes in the same gene systems noted in our strains include a frameshift mutation in *agrC*, a premature stop codon in *yycH*, and a frameshift mutation in *prsA* [54]. Other mutations not detected in strains CB1540 and CB1541 by us have been described in various VISA strains [53].

Recently, Shoji et al. [39] observed an association in mutations in *walKR*, *clpP*, *graRS*, and *vraSR* in VISA strains, with mutations in *walKR* being carried most frequently. Separate introduction of mutated *walK* or *clpP* into a VISA parent strain raised the vancomycin MIC from 1 to 2 µg ml^−1^, but introduction of both raised the MIC to 4 µg ml^−1^, and resulted in a thickened cell wall and decreased autolysis. Howden et al. [55] have emphasized the importance of mutations in *walKR* in the evolution of multidrug resistance in *S. aureus*. Single mutation in either *walK* or *walR* led to vancomycin and daptomycin co-resistance and cell wall thickening, which is typical of VISA. Similar functional studies would be necessary to prove that the mutation in *walK* we observed impacted daptomycin and vancomycin resistance.

### The Strain CB1540 Transcriptome

Compared to strain CB1118, 170 genes were increased in expression and 152 genes were decreased in expression two fold or more in strain CB1540 (Table S1). Considerable numbers of genes in the categories of cell envelope (although most were genes of the *cap* operon), cellular processes, metabolism, regulatory function, hypothetical proteins and proteins of unknown function were upregulated.

Various genes of the *cap* operon were strongly upregulated. The *cap* genes encode a microcapsule associated with virulence [56]. Over expression of *cap* genes has been shown in VISA isolates compared to their VSSA parent strains [18], [57], [58]. It seems possible that production of capsular polysaccharide material could impede the access of daptomycin to its target in the cytoplasmic membrane. However, the *cap* operon was not upregulated in strain CB1541.

In strain CB1540, several genes involved in the likely accumulation of the compatible solute glycine betaine were upregulated including *cudT* (SAV2615), a likely choline transporter, *betA* (SAV2612, choline dehydrogenase), and *gbsA* (SAV2613, glycine betaine aldehyde dehydrogenase). These genes are involved in the uptake and metabolism of choline to produce glycine betaine [59]. Also *opuD2*, glycine betaine transporter 2, SAV 2185, a glycine betaine transporter *opuD* homolog, and *proP*, a putative proline/betaine transporter were upregulated. *S. aureus* accumulates proline and glycine betaine in response to osmotic stress [60]. However, glycine betaine also has beneficial effects on protein structure in stressed cells [61], [62], and accumulation of glycine betaine may allow bacteria to be primed to withstand the stress of daptomycin challenge. Cell wall and membrane active antibiotics cause oxidative stress and protein aggregation and misfolding as revealed by the induction of molecular chaperones [63]–[65].

Genes encoding the surface proteins SdrD, ClfA and ClfB were upregulated. These proteins play a role in the ability of *S. aureus* to adhere to squamous epithelial cells [66].

Urease operon genes (*ure*) were upregulated. Ammonia and CO_2_ are produced through urease activity and ammonia production may serve to neutralize environmental acidity. Alternatively, urease induction may be necessary to detoxify excess urea that may be produced through possible changes in metabolism in the mutant.

*mscL* was upregulated 2.1 fold. This gene encodes a large conductance mechanosensitive channel that can relieve excess turgor pressure building up in bacterial cells [67]. Perhaps the upregulation of various genes involved in osmoregulation leads to higher levels of compatible solutes in the mutants and MscL relieves this turgor pressure that develops as needed. This gene was also upregulated in strain CB1541 (Table S2).

Genes encoding enzymes of the nitrate and nitrite reductase complexes (*narH*, *narE* and *narI*) that are increased in expression under anaerobic conditions were upregulated in strain CB1540.

Various transcriptional regulators were upregulated including *gntR*, a gluconate operon repressor; *sarH2*, staphylococcal accessory regulator U; *icaR*, the *ica* operon repressor; and *nirR*, transcriptional regulator of the *nir* operon.

Several genes encoding hypothetical proteins were strongly upregulated including SAV1509, 7.8 fold, MW2624, 9 fold; MW2454, 5.9 fold; MW0920, 5.2 fold; MW0523, 4.3 fold; SAV2205, 5.6 fold.

A significant number of genes were down regulated in expression in strain CB1540 in the categories of cell envelope, energy metabolism, protein synthesis, purines-pyrimidines nucleosides and nucleotides, transport and binding proteins, and hypothetical proteins.

SAV2515, encoding probable transmembrane protein SmpB, was down regulated 11.2 fold. *smpB* encodes the small protein B that binds to transfer-messenger RNA (tmRNA). It plays essential roles in rescuing the tmRNA molecule from degradation in the cell, enhancing the aminoacylation of tmRNA and mediating the binding of tmRNA to the ribosome [68]. It is also reported that tmRNA is required for the growth of *Bacillus subtilis* under stress [69]. The binding of SmpB to tmRNA is a critical component of trans-translation where incompletely synthesized proteins receive a peptide tag, an important aspect of quality control in protein synthesis. In the absence of SmpB peptide tagging is abolished [70]. The regulatory activities of certain repressor proteins are increased in tmRNA (*ssrA*)-defective cells and SmpB mutants mimic SsrA mutants. Decreased transcription of *smpB* may decrease cellular levels of SmpB and lead to altered regulation of gene expression.

Various genes involved in fermentation were downregulated including genes encoding lactate dehydrogenase (*lctE* and SAV 2602), *pflA* encoding pyruvate formate-lyase activation enzyme, and *pflB*, formate lyase.

Twenty three genes encoding proteins involved in protein synthesis were down regulated, including a considerable number of genes encoding ribosomal proteins. This is consistent with the slow growth of this strain.

Genes of both the purine and pyrimidine biosynthesis operons were significantly down regulated. Interestingly, strain CB1540 has a mutation is *prs*, phosphoribosyl synthetase. Perhaps this causes the strain to depend more on exogenous purines and pyrimidines.

*spa*, which encodes protein A (or precursor), was downregulated 3.7 fold. SAV0631 encoding a lipoprotein was down regulated 12 fold.

### The Strain CB1541 Transcriptome

In strain CB1541 242 genes were over-expressed and 222 genes were under-expressed 2 fold or more compared to strain CB1118 (Table S2). Significant number of genes in the categories of cellular processes, central intermediary metabolism, energy metabolism, protein fate, regulatory functions, transport and binding proteins and hypothetical proteins were changed in expression (Table S2).

The strain CB1540 and CB1541 transcriptomes had changes in expression of some genes in common but many differences were apparent.

*lytM* encoding glycyl glycine endopeptidase, a peptidoglycan hydrolase acting in the *S. aureus* interpeptide bridge, was upregulated 8 fold. *ssaA* was upregulated 9.3 fold. *ssaA* is a member of the *walK* regulon, as is *lytM*. Genes involved in undecaprenyl phosphate metabolism were upregulated (SAV0683 and *bacA*).

Like CB1540, *betA*, and *gbsA*, involved in the conversion of choline to glycine betaine were strongly upregulated. *opuCC* encoding a glycine betaine/carnitine/choline ABC transporter was upregulated, as was *treP* encoding a trehalose-specific PTS transporter. The possible accumulation of compatible solutes appears to be a component of daptomycin resistance in this strain also. *mscL* encoding a large-conductance mechanosensitive channel was upregulated as it was in strain CB1540.

There was stronger upregulation of *ureB-F* in strain CB1541 than in CB1540.

In contrast to strain CB1540 genes involved in glycolysis and fermentation tended to be upregulated.

Also, in contrast to strain CB1540, various stress protein genes were upregulated including *clpC*, *clpP*, *grpE*, *groES*, *clpX*, *groEL*, *dnaJ*, and *msrA*. These proteins are involved in correcting protein misfolding, and degrading terminally damaged protein. This is consistent with the mutation in *clpP* in that ClpP controls the expression of stress protein chaperone and protease genes, as revealed by their overexpression in a Δ*clpP* mutant [49].

SAV2095 encoding a protein similar to the *sceD* precursor was upregulated 16.9 fold. *sceD* encodes a putative lytic transglycosylases and inactivation of *sceD* resulted in impaired cell separation [71].

SACOL2139 and MW0934 encoding hypothetical proteins were upregulated 7.9 and 5 fold and, SAV0523 was overexpressed 6.1 fold.

Significant numbers of genes in the following categories were down regulated: cellular process, energy metabolism, regulatory functions, transport and binding proteins, and hypothetical proteins. *spa* was downregulated 10.2 fold, and SAV0631 lipoprotein 7.9 fold. MW1888 was downregulated 22.1 fold, MW2405 6.5 fold, MW2524 5.6 fold. Probable transmembrane protein *smpB* was highly downregulated (15.7 fold) in CB1541 as it was in strain CB1540.

The FemAB protein genes involved in peptidoglycan interpeptide bridge formation were downregulated. These genes were also down regulated in strain CB1540. Strain CB1540 also showed decreased autolysis and also may have a shortened interpeptide brige.

Other cell wall genes down regulated were antiholin protein gene *lrgB* and autolysin gene *atl*. *lrgB* is over expressed in response to daptomycin [11]. Mehta et al. [52] also observed *lrgB* was downregulated in the strains they studied. Downregulation of *lrgB* expression may be a way of decreasing responsiveness to daptomycin challenge.

*crtN* encoding squalene synthase involved in carotenoid biosynthesis was down regulated two fold consistent with the lower carotenoid content of strain CB1541.

Six genes from CB1540 and eight genes from CB1541 are randomly selected and their expressions are confirmed by RT-PCR (Table S4).

### Transcriptional Profiling Studies in Other Daptomycin-Resistant Strains And VISA

The first genome-wide transcriptional profiling study of a daptomycin-resistant strain versus a susceptible parent appears to be that of Camargo et al. [35]. These authors studied a laboratory-derived strain (MIC 3 µg ml^−1^). There was only limited similarity between their results and ours. However, as in our results, *opuD* and *mscL* were over-expressed.

Fischer et al [72] have reported both transcriptomic and proteomic studies of a clinical daptomycin-resistant (MIC 2 µg ml^−1^) and its susceptible parent strain. There was little similarity in the transcriptome of this strain compared to our data. There was significant upregulation of various *lac* genes, *lytN*, *lytH*, cell wall biosynthesis genes and virulence factor genes in the Fisher et al [72] study.

Mehta et al. [52] reported that *vraS* and *vraR* were over expressed in three pairs of daptomycin-resistant strains versus their susceptible parent strains. VraSR is the two-component system that controls a significant part of the cell wall stress stimulon [73]. We did not observe over expression of *vraS* and *vraR* in our strains.

Transcriptional profiling studies of VISA strains compared to VSSA parent strains have been reviewed by Howden et al. [53]. Overall, a variety of different transcriptomes have been observed in different VISA strains in different studies. Upregulation of *vraSR* and the cell wall stress stimulon, purine biosynthesis, *graSR* and genes controlled by this two-component system and various regulatory genes have been reported in some but not all strains. The most consistent transcriptional change noted is down-regulated expression of *spa* encoding protein A. *spa* transcription was decreased in strains 1540 and 1541 (Tables S1 and S2).

### Decreased Autolysis of Strains CB1540 and CB1541

**Figure 1:**
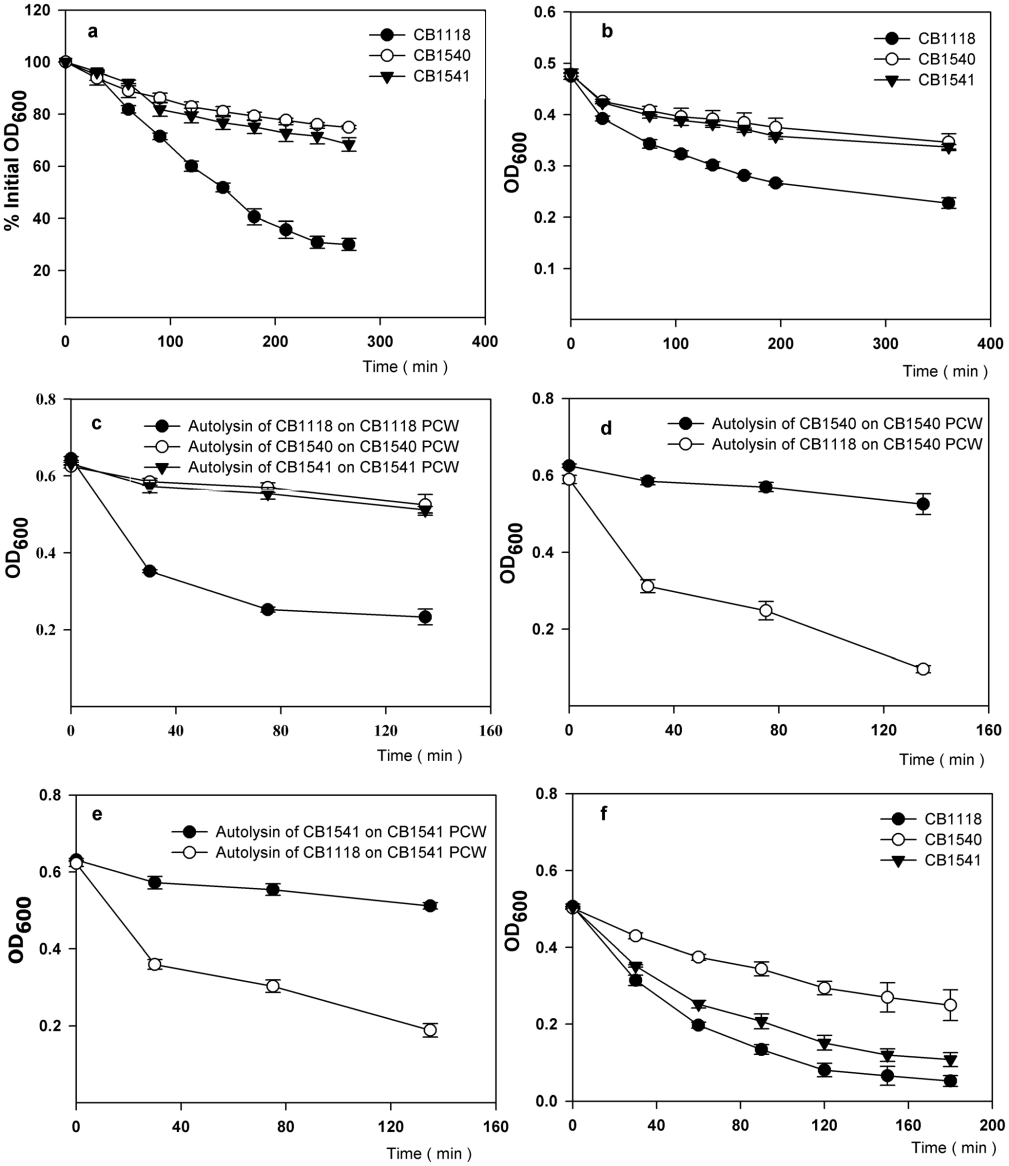
Autolysis and lysostaphin susceptibility of strains CB1118, CB1540 and CB1541. (a), Triton X-100 stimulated whole cell autolysis; (b), autolysis of isolated crude cell walls; (c), activity of autolysin extracts on purified cell walls (PCW); (d) and (e) activity of autolysin extracts on PCW of the strain that was source of the autolysin extract and activity of strain CB1118 autolysin extract on strain CB1541 and CB1540 PCW (f) lysostaphin digestion of PCW.

The Triton X-100 stimulated rates of autolysis of the parent and mutant strain are shown in Figure 1a. Both daptomycin-resistant strains showed markedly slower rates of autolysis than the parent strain. This is a common but not universal finding in VISA and daptomycin-resistant strains [15], [18], [36], [53], [74], [75]. This decreased rate of autolysis was also seen in crude cell walls retaining autolytic activity of strains CB1540 and CB1541 compared to those isolated from the parent strain that showed good activity (Figure 1b). This indicates that either the enzyme(s) autolysin, or the substrate, cell wall, or possibly both are changed in the daptomycin-resistant strains. In order to investigate this further we prepared freeze-thaw autolysin extracts from each strain and investigated their activities on their own purified cell walls that do not retain autolytic activity and on purified cell walls of the other strains. Freeze-thaw autolysin extract from strain CB1118 caused good lysis of its own purified cell walls (Figure 1c). However, freeze-thaw extracts of the daptomycin-resistant strains had negligible activities on their own purified cell walls (Figure 1c) or purified cell walls from strain CB1118 (data not shown). Nevertheless, purified cell walls from both strains 1540 and 1541 were readily digested by the freeze-thaw extract from strain CB1118 (Figure 1d and e). This indicates that the major reason for the decreased autolytic activity of strains CB1540 and CB1541 is due to decreased autolysin activity rather than a change in cell wall structure.

**Figure 2:**
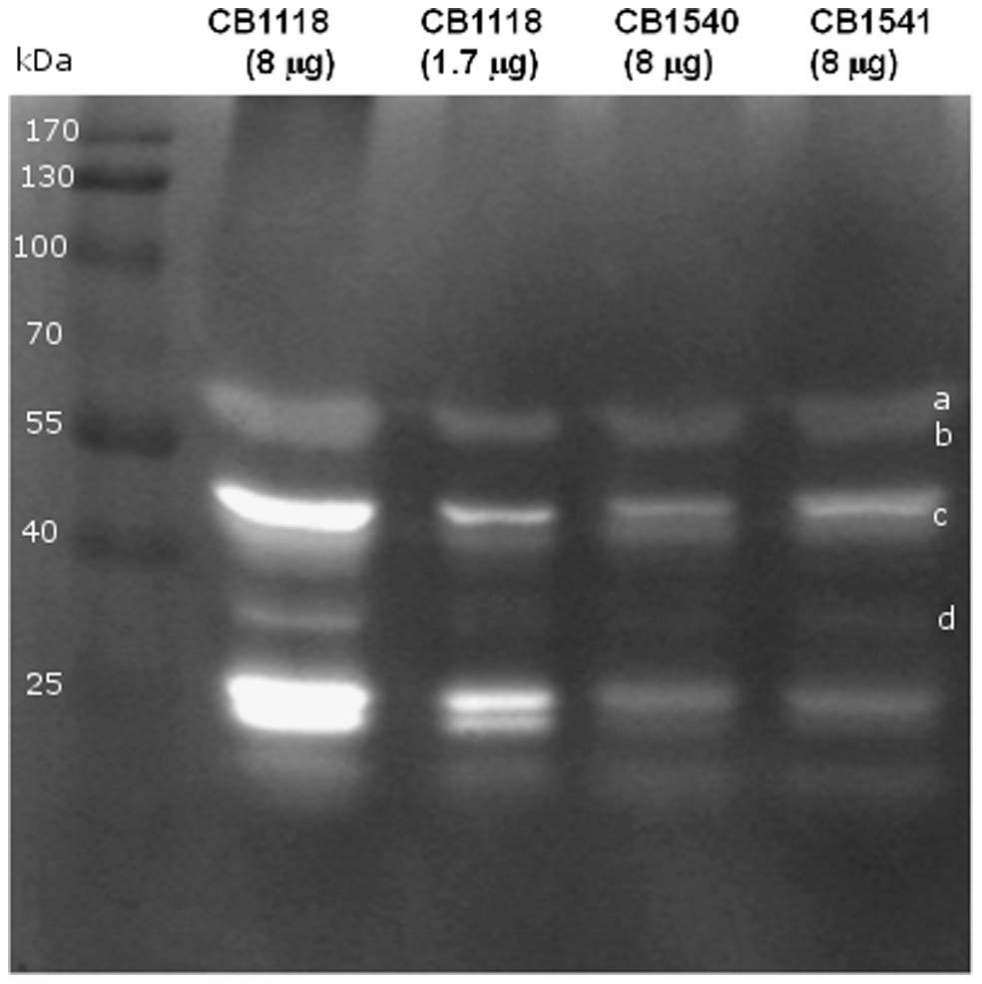
Zymographic analysis of autolysins. The strains from which the autolysin extract was prepared and amount of protein loaded are indicated on the top of the gels. Molecular mass markers are indicated in kilodaltons on the left side of the gel. a,b. Atl (62 kDa-51 kDa) c. LytN (46 kDa) d,. LytM (36 kDa).

This was investigated further by zymographic determination of the peptidoglycan hydrolase profiles of the strains (Figure 2). The extract from strain CB1118 showed a typical peptidoglycan hydrolase profile with multiple bands showing intense clearing of the substrate in the gel [75], [76]. In contrast, the freeze-thaw extracts from the resistant strains were much less active when the same amount of protein was loaded on the gels (Figure 2), supporting the idea that there is less autolysin or less active autolysin, assuming that the freeze-thaw extraction is equivalent in all three strains.

The purified cell walls of strains CB1540 and CB1541 were less susceptible to lysostaphin digestion than those of strain CB1118, with strain CB1540 walls being the least susceptible (Figure 1f). Because lysostaphin targets the glycylglycine interpeptide bridge this may indicate some changes in the interpeptide bridge in the mutants, which is compatible with changes in expression of *fem* and *femH* genes that are involved in biosynthesis of the glycine interpeptide bridge. Patel et al [24] reported decreased autolysis and decreased lysostaphin susceptibility in some of the daptomycin-resistant strains they studied. Their strains also had mutations in *walK*, as did strains CB1540 and CB1541. Functional studies of the same mutation in parent strain CB1118 would be necessary to establish our *walK* mutation as contributing to decreased autolysis. Bertsche et al. [77] compared the cell wall composition of datomycin-resistant strains to susceptible parent strains. In the resistant strains they found thicker cell walls, decreased peptidoglycan O-acetylation, and increased amounts of total wall teichoic acid that also showed increased D-alanylation. The decreased autolytic activity of our daptomycin-resistant strains is very similar to the situation described in VISA where decreased production or activity of autolysin enzymes is responsible for decreased autolytic activity [74]. The major autolysin of *S. aureus* is Atl [78] and we did observe some decreased expression of *atl* in strain CB1541 (Table S2). In contrast *lytM* was increased in expression 8 fold in strain CB 1541 but the zymographic examination did not reflect increased expression of LytM. However, the regulation of the autolytic pathway is complex and involves proper localization of autolysin in the cell wall and proteolytic processing of autolysins [75], [76], [79], and further work will be necessary to understand the autolysin deficiency in more detail.

Nevertheless, decreased autolysis is likely to increase the ability of bacteria to survive the bactericidal effects of cell envelope-active antibiotics like daptomycin and vancomycin [75], [80] Since WalKR plays an important role in autolysin regulation the mutation in *walK* may well be involved in decreased autolysis [31], The *agr*-negative phenotype of the strains may also be involved in decreased autolysis [81].

### Ultrastructure of the Strains

**Figure 3:**
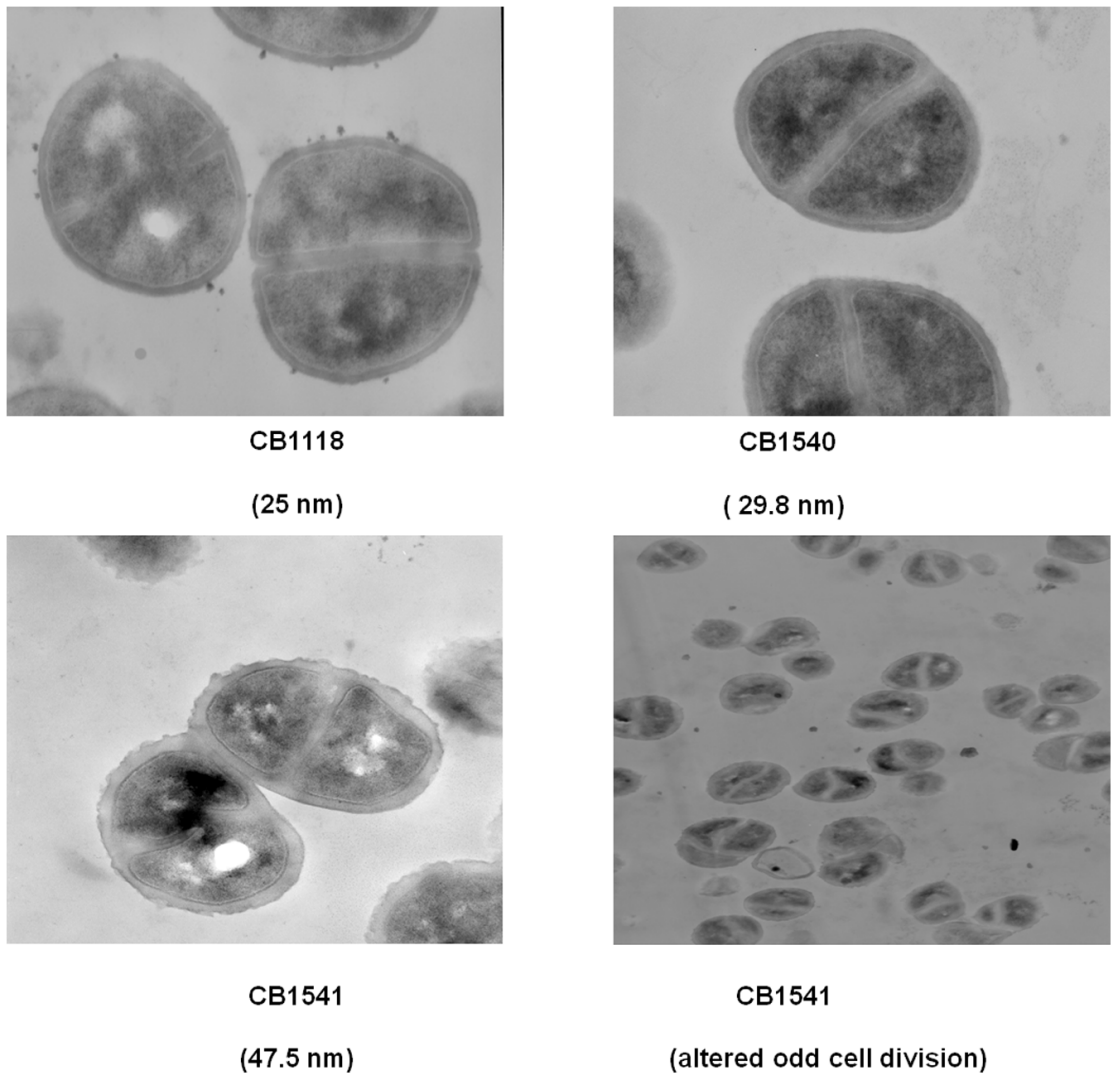
Ultrastructure of strains CB1118, CB1540 and CB1541. Magnification 50,000×.

Strain CB1541 had walls that were about twice as thick as those of the parent strain-48 nm versus 25 nm. Strain CB1540 walls were slightly thicker than those of the parent strain (Figure 3). Thickened cell wall is a common finding in daptomycin-resistant strains [36] and it is thought that the thickened cell wall might reduce the access of daptomycin to its target in the cytoplasmic membrane. However, some daptomycin-resistant strains do not show thicker cell walls [36]. In addition, misplaced division septa were seen in strain CB1541, the strain with the thickest cell walls (Figure 3). Shoji et al. [39] have reported that mutations in both *walK* and *clpP* lead to thickened cell walls. Both strains CB1540 and CB1541 had a mutation in *walK* and strain CB1541 also had a mutation in *clpP*.

### Carotenoid Content And Fatty Acid Composition of Daptomycin-Resistant Strains

The total carotenoid content of the strains was measured as OD_465_ of warm methanol extracts [82], [83] (Table 1) and indicated that strains CB1540 and CB1541 had lower cell carotenoid contents than the parent strain. Decreased carotenoid content is consistent with the white color of CB1540 and CB1541 colonies.

Jones et al. [22] have provided evidence that increased membrane fluidity is associated with daptomycin resistance. The major determinant of membrane fluidity is the fatty acid composition of the phospho- and glyco- glycerol membrane lipids [84]. The fatty acid compositions of the strains are shown in Table S1. The fatty acid composition of strain CB1541 was consistent with increased membrane fluidity compared to that of parent strain CB1118. Straight chain fatty acids were decreased to 20.7% from 27.8%, iso fatty acids to 15.5% from 19.2% and anteiso fatty acids increased to 63.9% from 52.6%. The anteiso:iso fatty acid ratio was 4.1 in strain CB1541 compared to 2.7 in CB1118. Both a decrease in carotenoid content and an increase in anteiso fatty acids, are expected to result in increased membrane fluidity [84], [85], and this is in line with previous reports of an association between decreased susceptibility to daptomycin and higher membrane fluidity [22], [29]. The fluorescence depolarization values for the strains were: CB1118, 0.31±0.02; CB1540, 0.32±0.015; CB1541, 0.28±0.005, indicating that the membrane of strain CB1541 is more fluid than those of the other two strains. The fatty acid compositions of strains CB1540 and CB1118 were similar to each other (Table S3).

## Summation

Mutations in a considerable number of different genes have been described in various laboratory and clinical daptomycin-resistant strains. Mutations in phospholipid biosynthesis genes have been commonly reported and these mutations may well alter the interaction of daptomycin with its target, the membrane. However, we did not observe any mutations in phospholipid biosynthesis genes, although we did observe changes in fatty acid composition and carotenoid content compatible with a more fluid membrane that has previously been reported to be associated with daptomycin resistance. In clinical MRSA isolates, Mishra et al. [86] have hypothesized that the course of development of daptomycin resistance could be influenced by in vivo exposures to endogenous host defense peptides. In our strains we observed mutations in *walK* and *agrA*. Both these systems appear to be involved in control of autolytic activity. The transcriptomes of the two resistant strains were different from each other. The transcriptomes indicated significant changes in metabolism and changes in display of cell surface proteins. Both strains appeared to be primed to respond to daptomycin stress, which likely causes the accumulation of damaged proteins, in that genes involved in accumulation of compatible solutes were upregulated in both strains, and heat shock chaperones and proteases genes were upregulated in one strain. Both strains had much less autolytic activity in freeze-thaw extract than the parent strain. Decreased autolysis has been associated with tolerance to the killing action of bactericidal antibiotics. We think this is important along with the slow growth of the mutants, which is also associated with decreased antibiotic susceptibility. Mutations in WalKR indicate *walKR* may be one of a limited number of genes key for the development of daptomycin and vancomycin resistance, such that mutations in phospholipid biosynthesis genes may be unnecessary for resistance development.

## Materials and Methods

### Strains Studied and Growth Conditions

The strains studied are described in Table 1. The daptomycin-resistant strains CB1540 and CB1541 were obtained by serial passage of strain CB1118 (MW2, community-associated MRSA, kindly provided by B.N. Kreiswirth), in the laboratory with increasing concentrations of daptomycin as described previously [30]. The serial passage was set up as a series of MICs using 3 different high test concentrations (20, 24 and 32 ug/ml). In order to detect incremental changes in DAP susceptibility, an extended gradient was created by combining the three sets of two fold dilutions. 104 colony forming units (CFUs) of exponentially growing cells were used as inocula into the first day's MIC plates. After approximately 18 hours of growth at 37°C, the MIC was determined as the highest concentration that permitted growth and then diluted 1∶1000 to inoculate the next day's plates containing the same extended DAP gradient. The process was repeated for 20 days, giving rise to CB1540 and CB1541 (as independently derived from CB1118). Stability of the mutants was established by maintenance of daptomycin MIC after four days or more of transfer on drug-free medium. Strains CB1540 and CB1541 are independent day 23 isolates. The strains were grown in 50 ml Mueller-Hinton broth (Difco) supplemented with CaCl_2_ (50 ug ml^−1^) in a 250 ml Erlenmeyer flask at 37°C with shaking at 210 rpm. Cultures were inoculated with 0.5 ml of an overnight culture. For transcriptional profiling the strains were grown to an OD_600 nm_ of 0.4.

### Transmission Electron Microscopy

Preparation and examination of *S. aureus* cells by transmission electron microscopy were performed as described previously [87]. Thin sections stained with uranyl acetate and lead citrate were examined in a Zeiss 10-C transmission electron microscope operating at 60 kV. Cell-wall thickness measurements were performed using photographic images at 50,000× final magnification. Thirty five cells of each strain with nearly equatorial cut surfaces were measured, and results were expressed as mean value ± standard deviation.

### Complete-Genome Comparisons (CGS)

These were performed with an array-based service (CGS) provided by Nimblegen Systems Inc (Madision, WI) [30], [88]. The reference genome was *S. aureus* MW2 (CB1118), which was compared to those to those of strains CB1540 and CB1541.

### PCR Amplification and DNA Sequencing

CGS-identified single nucleotide changes were confirmed by sequencing of PCR-amplified regions covering each putative nucleotide mutation. The PCR primers used are shown in Table 3.

**Table 3:** PCR primers.

Genes	Primers 5′-3′
*walK*	Forward: ACTTTGGCGATGTACGTACG
	Reverse: AGCCCGATAATTTGCATACC
*prs*	Forward: TAAACGTCGTCCTAGACCAAATG
	Reverse: TGCTTGTGCAGCTAAAGTGA
MW1109	Forward: GGATTTTGTAAAAATATTGAAAGTGAA
	Reverse: CCTTTACCGCCACTATCAACA
*pnpA*	Forward: AAGAAATCGTCAATGAATTTATCGA
	Reverse: CAATTTCACGACGACCTGG
*agrA*	Forward: ACTGATAATCCTTATGAGGTGC
	Reverse: AAATAAAATCCATCGCTGCAA
MW1927	Forward: TGTTGCGATACTTGCGAATC
	Reverse: ATTCGTCGATGAGCATTTCA
*clpP*	Forward: CAAGCGCAAGACTCAGAGAA
	Reverse: TGCAGCAATTTCGATTTCAG
MW1913	Forward: CTATCATTTCACGTATTTCTTTCC
	Reverse: AACAAGAGGAGGAGATTTAAATGATG

### Transcriptional Profiling

This was carried out essentially as described by Muthaiyan et al. [11]. Total bacterial RNA was obtained using RNA protect solution and breaking cells using the FastPrep system. Purified labeled cDNA was hybridized with *S. aureus* genome microarrays version 6.0 provided by the Pathogen Functional Genomics Resource Center (PFGRC) of the National Institutes of Allergy and Infectious Diseases (NIAID). The full genome array consists of 16024 70-mer oligonucleotides representing 4546 ORFs from *S. aureus* strain COL, MW2, MSSA476, Mu50, MRSA252, MSSA476, N315, USA300-FPR3757,and pLW043. Each ORF is printed in four times on the array. Hybridization signals were scanned, analyzed using TIGR spotfinder, normalized applying the LOWESS algorithm using TIGR, MIDAS software. The normalized log2 ratio of test to reference signal for each spot was recorded. Genes with fewer than three data points were considered unreliable, and their data points were discarded. The averaged log2 ratio for each remaining gene on the six replicate slides was ultimately calculated. Significant changes in gene expression were identified with SAM (significance analysis of microarrays; http://www-stat.stanford.edu/~tibs/SAM/) software using one class mode. SAM assigns a score to each gene on the basis of change in gene expression relative to the standard deviation of repeated measurements. For genes with scores greater than an adjustable threshold, SAM uses permutations of the repeated measurements to estimate the percentage of genes identified by chance, i.e., the false discovery rate. A cutoff of 2-fold for over- and underexpressed ORFs was used. To examine how genes with transcript level changes are distributed with regard to their function, we further classified these genes using our in-house software Gene Sorter according to the categories described in the comprehensive microbial resource of TIGR (http://cmr.tigr.org/tigr-scripts/CMR/shared/Genomes.cgi). Several controls were employed to minimize the technical and biological variations and to ensure that the data obtained were of good quality. First, each ORF was present in triplicate on the array. Second, each RNA preparation was used to make probes for at least two separate arrays for which the incorporated dye was reversed. Finally, three independent cultures were used to prepare RNA samples.

### Microarray Data Accession Number

The data discussed in this publication have been deposited in NCBI's Gene Expression Omnibus (GEO) (http://www.ncbi.nlm.nih.gov/geo/) and are accessible through GEO Series accession number GSE40753.

### Microarray Validation By Real-Time Reverse Transcription-PCR (RT-PCR)

To confirm the validity of microarray data, gene specific mRNAs were quantified from resistant strains and parent strains by quantitative real-time PCR. Total RNA preparation was performed exactly as described for the microarray assay. Residual DNA was removed from the samples by performing on-column DNase digestion step with RNase-free DNase (QIAGEN). cDNA was synthesized using high capacity RNA-to-cDNA kit (Applied Biosystems, Foster City, CA). Specific primers for the genes tested and for 16S rRNA, were designed using Primer Express software (Applied Biosystems) in order to design 100- to 200-bp amplicons (Table 4). Real-time PCR was performed with the ABI PRISM 7300 sequence detection system (Applied Biosystems) and SYBR green technology. Quantitative real-time PCRs (qRT-PCRs) were performed in a 25-µl reaction volume containing 1 µl of a 1/100 dilution of cDNA, 1 µl of gene-specific primers (10 µM), and 12.5 µl of Power SYBR Green PCR Master Mix (Applied Biosystems, Foster, CA). The expression levels of the tested genes were normalized using the 16S rRNA gene of *S. aureus* as an internal standard whose transcript level did not vary under our experimental conditions. Each assay was performed in triplicate and repeated with at least three independent RNA samples. No-template reactions were included as negative controls. A negative control without reverse transcriptase in the reaction was performed to exclude the possibility of DNA contamination. All samples were amplified in triplicate and the data analysis was carried out using 7300 System software (Applied Biosystems).

**Table 4:** Primers for RT-PCR.

Genes	primers 5′-3′	
*16s*	Forward	CCACGCCGTAAACGATGAGT
	Reverse	CACATGCTCCACCGCTTGT
*bet*	Forward	GTTAAAACGAGGGCCAGCAA
	Reverse	ATTCGGCGACCACGATGTAC
*gbsA*	Forward	GGGTTGAAAGCGCGAATAAA
	Reverse	CTTGCGACCATTCACCAGACT
*mscL*	Forward	GGGTGCAGCTTTCAACAAGATT
	Reverse	AAGTCGATAACAGATTGGATAAATAAACC
*ftsH*	Forward	TCCTCCGCCAAGCACAAGGT
	Reverse	TGCAACCGCTCTAGCAAGTA
*yycG*	Forward	CAATCACCGATATGCGTAACCA
	Reverse	CAGTATTAGCCTGCGCTTCTTG
*lytM*	Forward	AGGTCCAGACGCGAGCTATTAT
	Reverse	TCTTTCGCATGACCACTAGCTG
*lrgA*	Forward	TCATTTATGCCAATTCCTATGCCT
	Reverse	ACCGGCTGGTACGAAGAGTAAG
*lrgB*	Forward	TTGGCATCGTATCATCGGAG
	Reverse	GATACTGGTAACGCAATCGCTG
*accA*	Forward	TCGCTTCGGGTGTTGATATT
	Reverse	TTACGCCTGCTTCAACCTTT
*ftsA*	Forward	TCGCTTCGGGTGTTGATATT
	Reverse	TTACGCCTGCTTCAACCTTT

### Autolysis, Preparation of Cell Walls And Peptidoglycan Hydrolase Profiles

Triton X-100 stimulated autolysis of whole-cells grown in MHB was determined as described by Gustafson et al. [89]. Crude cell walls retaining autolytic activity and purified cell walls, which have been digested with trypsin and do not retain autolytic activity, were prepared as described previously [75], [90]. Freeze-thaw autolysin and determination of peptidoglycan hydrolase profiles by zymography were carried out as described by Koehl et al. [75].

### Fatty Acid Composition

Washed exponential phase cells were saponified and methylated and analyzed for fatty acid composition on an Agilent 5890 dual-tower gas chromatograph. Fatty acids were identified using the MIDI microbial identification system (Sherlock 4.5 Microbial identification system) at Microbial ID (New York, DE) [91].

### Total Carotenoid Determination

This was determined by extracting cells with warm methanol by the method of Morikawa et al. [82] as described by Davis et al. [83].

### Measurement of Membrane Fluidity

This was determined as described previously [92]. In brief, mid exponential phase cells (OD_600_ 0.6) were washed twice with 0.85% NaCl. Then cells were resuspended in 0.85% NaCl containing 2 uM 1,6-diphenyl-1, 3, 5-hexatriene (DPH; Sigma, MO) to an OD_600_ of 0.3 and incubated at 30°C for 1 h. 1 mM DPH solution was prepared in tetrahydrofuran, and 200 µl was added to 50 ml of 0.85% NaCl. DPH fluoresces in the hydrophobic regions of the lipid bilayer but does not fluoresce in an aqueous environment [93]. Excess tetrahydrofuran was removed by flushing with nitrogen. Fluorescence polarization was measured using QuantaMaster™40 spectrofluorometer (Photon Technology International, Inc., NJ). The excitation and emission wavelength for DPH were 360 and 426 nm respectively. The higher the value is the lower the membrane fluidity. The experiment was performed twice, and the mean polarization values were compared for significant differences by using the t test.

## Supporting Information

Table S1 **Expression of genes of daptomycin resistant strain CB1540 compared to strain CB1118.**
(XLSX)

Table S2 **Expression of genes of daptomycin resistant strain CB1541 compared to strain CB1118.**
(XLSX)

Table S3 **Fatty acid compositions of CB1118, CB1540 and CB1541.**
(XLSX)

Table S4 **Confirmation of gene expression changes by RT-PCR.**
(XLSX)
